# The Presence of Plasmids in *Lactococcus lactis* IL594 Determines Changes in the Host Phenotype and Expression of Chromosomal Genes

**DOI:** 10.3390/ijms24010793

**Published:** 2023-01-02

**Authors:** Katarzyna Kosiorek, Anna Koryszewska-Bagińska, Marek Skoneczny, Lidia Stasiak-Różańska, Tamara Aleksandrzak-Piekarczyk

**Affiliations:** 1 Institute of Biochemistry and Biosciences, Pawinskiego 5a, 02-106 Warsaw, Poland; 2 Department of Medical Biology, Medical University of Warsaw, Litewska 14/16, 00-575 Warsaw, Poland; 3 Department of Food Technology and Assessment, Institute of Food Sciences, Warsaw University of Life Sciences-SGGW, Nowoursynowska 159C St., 02-787 Warsaw, Poland

**Keywords:** *L. lactis* IL594, *L. lactis* IL1403, plasmid, phenotypic and transcriptomic microarrays

## Abstract

The *L. lactis* IL594 strain contains seven plasmids (pIL1 to pIL7) and is the parental strain of the plasmid-free *L. lactis* IL1403, one of the most studied lactic acid bacteria (LAB) strain. The genetic sequences of pIL1 to pIL7 plasmids have been recently described, however the knowledge of global changes in host phenotype and transcriptome remains poor. In the present study, global phenotypic analyses were combined with transcriptomic studies to evaluate a potential influence of plasmidic genes on overall gene expression in industrially important *L. lactis* strains. High-throughput screening of phenotypes differences revealed pronounced phenotypic differences in favor of IL594 during the metabolism of some C-sources, including lactose and β-glucosides. A plasmids-bearing strain presented increased resistance to unfavorable growth conditions, including the presence of heavy metal ions and antimicrobial compounds. Global comparative transcriptomic study of *L. lactis* strains revealed variation in the expression of over 370 of chromosomal genes caused by plasmids presence. The general trend presented upregulated energy metabolism and biosynthetic genes, differentially expressed regulators, prophages and cell resistance proteins. Our findings suggest that plasmids maintenance leads to significant perturbation in global gene regulation that provides change in central metabolic pathways and adaptive properties of the IL594 cells.

## 1. Introduction

Lactococci belong to lactic acid bacteria (LAB), which are considered the most numerous group of bacteria associated with human. This group encompasses species important in fermented food production, among which *Lactococcus lactis* is one of the most widely applied starter cultures, especially in cheese making [1]. Moreover, the recent evaluation of the potential use of *L. lactis* strains as a delivery vehicle for vaccines and therapeutic peptides only increase the importance of the already extensive body of lactococcal research [2,3]. 

Lactococcal strains mainly inhabit two natural environments: milk and plants, of which plants appear to be the primary niche, where adaptation to the nutrient-rich dairy habitat has resulted in loss of some functions, leading to reduced chromosomes, an increase in a number of pseudogenes and acquisition of genes (often plasmid ones) vital for growth in milk. In the result of this adaptive evolution, the vast majority of diary-associated isolates are particularly abundant in plasmids compared to the relatively low number of plasmids among lactococcal strains isolated from non-dairy niches [4,5]. 

Plasmid vary in size, function and distribution. Dairy lactococci harbor between one and twelve plasmids [5], ranging in size from 0.8 to 193 kb (retrieved from https://www.ncbi.nlm.nih.gov/genome/, accessed on 11 July 2022). Lactococcal plasmids often carry genes that may be essential for survival under extreme conditions, but more importantly, these extra-chromosomal genetic elements enable adaptation to the nutrient-rich environment of milk. In fact, numerous functions of lactococci necessary for efficient fermentation, such as the utilization of lactate, citrate, lactose, oligopeptides, and ion transport are carried in plasmids [4]. Interestingly, of the twelve plasmids present in one strain of *L. lactis*, no less than ten carry putative genes critical for efficient growth in a dairy environment [5]. Comparative analyses of plasmid-encoded functions performed on a larger number of *L. lactis* strains confirmed the abundance of genes of biotechnological importance in their plasmid load [3]. In addition, genes involved in bacteriocin production, phage resistance, conjugation and exopolysaccharide synthesis are also included in the plasmid-encoded *L. lactis* repertoire [5]. Consequently, there is considerable selection pressure on dairy strains to retain these plasmids, since plasmid-free derivatives grow poorly in milk. Because plasmids are mobile elements, they can be readily exchanged between different strains by various mechanisms (through conjugation, transduction and transformation) [5] Acquiring plasmids can benefit bacteria not only by providing new traits through the metabolic genes they carry, which are key to their eventual industrial use, but also, as single studies indicate, by affecting the expression of chromosomal genes involved in metabolism and energy production [6]. Despite the fact that plasmids are an important component of the bacterial genome, the potential impact of plasmidic genes on overall gene expression is still poorly understood.

*L. lactis* subsp. *lactis* IL594 is the parental strain of the best studied representative among LAB, the plasmid-free *L. lactis* IL1403 derivative. *L. lactis* IL594 was originally isolated from a cheese starter culture and contains seven plasmids, named pIL1-pIL7. The *L. lactis* IL594 plasmidome has been fully sequenced, revealing a possible significant role of plasmid-encoded traits in the environmental adaptation of these bacteria [7]. However, only a small number of lactococcal plasmids presented in the literature have been functionally studied for their possible impact on the phenotype of host cells. Given the industrial importance of lactococcal plasmids, our study focused on the global changes in phenotypes and chromosomal gene expression levels caused by the presence of pIL1-pIL7 plasmids in *L. lactis* IL594. We hypothesized that the repertoire of plasmid-related features may lead to adaptive and industrially important metabolic traits in *L. lactis* IL594. Indeed, the results obtained indicate that the presence of the plasmid pool leads to an overall improvement in the metabolic fitness of the host, understood as subtly increased tolerance to a range of antimicrobial compounds, as well as improved metabolic capacity. Importantly, we also show here that these gained activities may result not only from the direct action of plasmid genes, but also from the activity of chromosomal genes as the effect of plasmid-chromosome cross-talk. In addition to the obvious cognitive purposes, a deeper knowledge of gene functions and interaction mechanisms may be important in modulating biotechnological fermentation processes provided by lactococcal strains.

## 2. Results

*L. lactis* IL594 has seven low to medium copy number plasmids. *L. lactis* IL594 is the parental strain of plasmid-free *L. lactis* IL1403 and contains seven plasmids (pIL1-pIL7) [7]. In our study, we determined the copy number of each plasmid (pIL1 to pIL7) in the cell using the qPCR technique. The amplification efficiency of control and target genes was linear (R^2^ = 0.9953). From the comparison of amplification efficiency, it was calculated that the number of copies per chromosome equivalent to pIL1, pIL2, pIL3, pIL4, pIL5, pIL6, and pIL7 were 2, 1, 2, 1, 2, 2, and 9, respectively.

**Figure 1:**
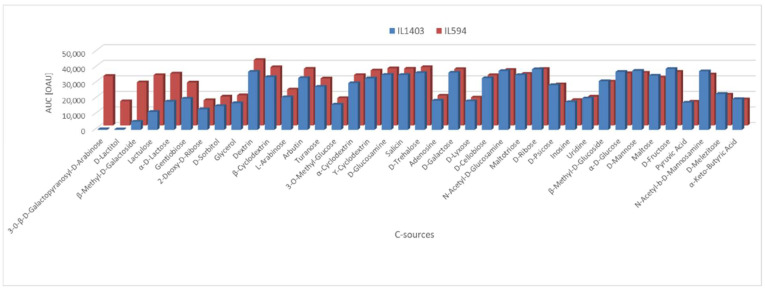
PM analysis for the utilization of carbon sources by *L. lactis* strains IL594 and IL1403. The height of the bars indicates the value of the area under the curve (AUC) of metabolic activity of the strains on a given C-source. Bars shown refer only to carbon sources for which metabolic activity of at least one strain was detected and the colorimetric reaction kinetics plot had an AUC value > 15,000 in Omnilog Arbitrary Units (OAU).

Carbon source utilization is reduced in *L. lactis* IL1403. The utilization by *L. lactis* IL594 and IL1403 of various carbon sources was analyzed in a global approach by two methods (API 50 CH and PM assays). Of the 49 carbohydrates present in API 50 CH, both strains were able to ferment only 14 of them. The fermentation efficiency of most of them (D-fructose, D-galactose, D-glucose, D-maltose, D-mannose, D-ribose, D-trehalose, N-acetylglucosamine, arbutin, cellobiose, and salicin) was the same for both *L. lactis* strains, whereas the metabolism of the other three carbohydrates (esculin, gentiobiose, and lactose) was increased in *L. lactis* IL594 compared to IL1403 Among the 190 different carbon sources present on the PM1 and PM2 panels (amino acids, carbohydrates, β-glucosides, sugar alcohols, amides, amines, esters, fatty acids, or carboxylic acids), for 39 of them at least one lactococcal strain showed notable (area under the curve [AUC] above 1.5 × 10^4^ in Omnilog Arbitrary Units [OAU]) metabolic activity. The most pronounced phenotypic differences in favour of IL594 were noted during the metabolism of C-sources such as 3-0-β-D-galactopyranosyl-D-arabinose, D-lactitol, β-methyl-D-galactoside, lactulose, D-lactose and gentiobiose, with particular emphasis on the first two substrates, which were not used at all by the plasmid-free strain. On the other carbon sources, the differences in metabolism between IL1403 and IL594 were far less significant or absent (Figure 1).

*L. lactis* IL594 has increased tolerance to a range of chemicals. To test the possibility that plasmids also affect the tolerance of IL594 to various chemical compounds, we used PM13-15 plates containing 72 chemical agents (e.g., antibiotics, chelators, nucleic acid analogs, oxidizing agents, toxic ions) at four different concentrations. The concentrations of compounds on the PM plates are proprietary to their manufacturer (Biolog Inc., Hayward, CA, USA). The magnitude of the concentration increment in the four PM wells is also unknown for each compound. Thus, in the following descriptions, we do not quantify (in µg/mL) the value of the levels of resistance to a given chemical, but instead use arbitrary units (OAU), which correspond to colorimetric changes in the metabolic activity of the strains, and focus on reporting the variations in tolerance to a given chemical between plasmid and plasmid-free strains. Overall, the multiplasmid strain showed increased tolerance to several chemicals compared to its plasmidless derivative. However, in each case there was no drastic difference in metabolic activity between the two strains as, at most and in a few cases only, ca. a 25% increase in the AUC of IL594 compared to IL1403 was observed.

**Figure 2:**
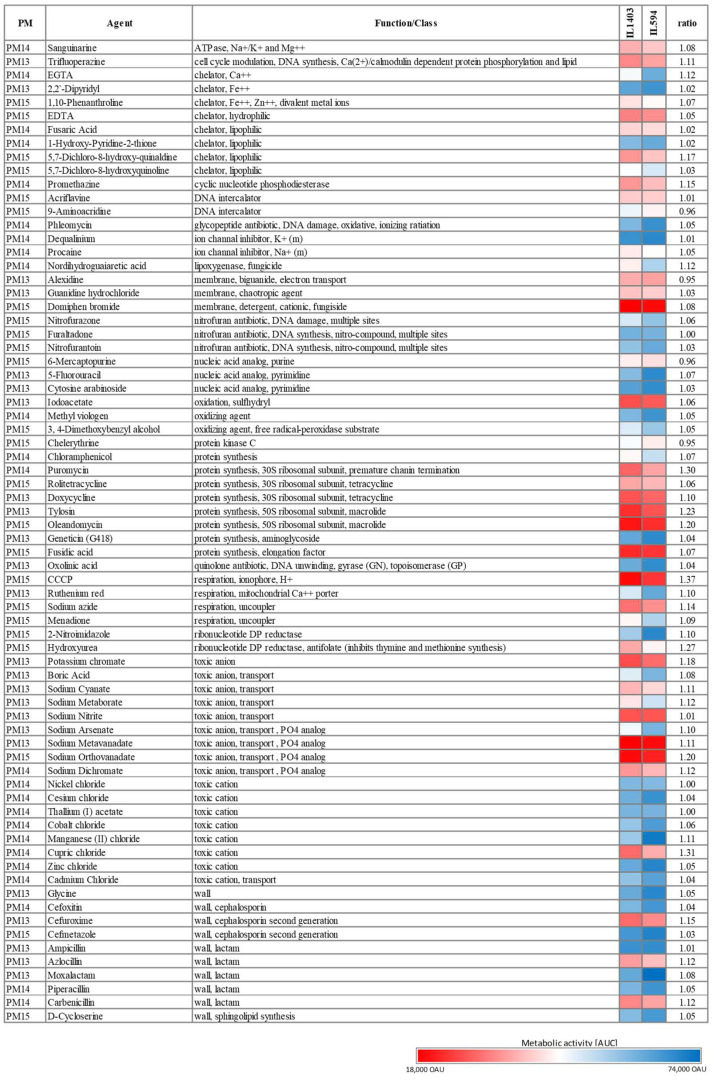
Tolerance of *L. lactis* IL1403 and IL594 to various chemical compounds. Colours indicate mean values of the areas under the curve (AUC) of the metabolic activity of IL1403 and IL594 recorded at four different compound concentrations. The highest average metabolic activity recorded was 74,000 OAU (Omnilog Arbitrary Units) and the lowest 18,000 OAU. The ratio was calculated as the quotient of the metabolic activity of IL594 and IL1403.

Among antibiotics tested, IL594 and IL1403 were tolerant to all concentrations of several β-lactams (ampicillin, moxolactam, piperacillin), aminoglycosides (geneticin), cephalosporins (cefoxitin, cefmetazole), glycopeptides (phleomycin), nitrofurans (nitrofurantoin, nitrofurazone, furaltadone), quinolones (oxolinic acid), and chloramphenicol. On the other hand, IL594 and IL1403 showed general sensitivity to macrolides (oleandomycin and tylosin), tetracyclines (doxycycline, rolitetracycline), aminonucleosides (puromycin) and fusidic acid. Among the cephalosporins and β-lactams, only cefuroxime, azlocillin and carbenicillin significantly inhibited the growth of the two strains. In the vast majority of cases, the presence of plasmids had no effect on the antibiotic resistance of *L. lactis*; such an effect was observed only for carbenicillin, azlocillin, cefuroxime, oleandomycin, tylosin, and puromycin, in the presence of which the multiplasmid strain performed slightly better (by 10–23%) than its plasmid-cured derivative (Figure 2).

Both IL594 and IL1403 strains showed full metabolic activity in presence of several toxic ions, particularly cations such as to the boron (Tl^+^), transition (Mn^2+^, Co^2+^, Cd^2+^, Ni^2+^, Zn^2+^) and alkali (Cs^+^) metals, whereas cupric chloride as well as most of the anions tested [m- and o-vanadate (VO_3_^−^ and VO_4_^3−^), chromate and dichromate (CrO_4_^2−^ and Cr_2_O_7_^2−^), nitrite (NO_2_^−^), metaborate (BO_2_^−^), cyanate (OCN^−^)] inhibited the metabolic activity of both strains to different extents. The presence of plasmids in some cases increased the resistance of the IL594 strain compared to its plasmid-free derivative. This was particularly pronounced with respect to such compounds as manganese (II) chloride, cupric chloride, sodium ortho- and metavanadate, sodium dichromate, sodium cyanate, sodium metaborate or potassium chromate, in the presence of which an approximately 10–20% increase in the metabolic activity of the parental strain was observed (Figure 2).

Metabolic activity coefficients were also generally slightly elevated for most other compounds in IL594 compared to IL1403. This was especially true for several chelators (e.g., EGTA and 5,7-dichloro-8-hydroxy-quinaldine), although most were lethal to both strains. IL594 was also more resistant to an NDGA lignan (nordihydroguaiaretic acid) and a variety of chemical compounds, including inhibitors of thymine and methionine synthesis (hydroxyurea), selected alkaloid derivatives (chelerythrine) and sodium azide (Figure 2).

The presence of plasmids in *L. lactis* IL594 affects the expression of chromosomal genes. To verify influence of plasmids on chromosomal gene expression (plasmid-chromosome crosstalk), the transcriptomes of IL594 and IL1403 were quantified by oligonucleotide microarrays. For this analysis, bacteria were cultivated in three growth conditions based on the type of sugar and its role in CcpA-dependent catabolic repression–repressive (glucose) and weakly-repressive (galactose) and non-repressive (cellobiose). Both strains grew comparably well in media containing each of these sugars, with a slight difference in growth rate in favor of strain IL1403 (cellobiose or glucose) or IL594 (galactose) (Supplementary Figure S1). For each condition, RNA was isolated from four time points, each representing a different phase of bacterial culture growth (early exponential [E], mid-exponential [M], transition [T], and stationary [S]). In this respect, cellobiose-supplemented medium appeared as the most differentiating culture condition as in this case, 382 changes in chromosomal genes expression were detected with 238 and 144 differences regarding up-regulation and down-regulation, respectively (Table 1). When strains were cultivated on other carbon sources, fewer changes in gene transcription were observed (232 and 129 for galactose and glucose, respectively). The strongest influence of plasmids presence on chromosomal genes expression occurred mainly in the exponential phases of growth (E and M), while the effect in the stationary phase was significantly reduced to a maximum of 51 differences in chromosomal genes expression depending on the sugar used (Table 1). The presence of plasmids more often activated than inhibited chromosomal gene expression (Table 1), and although there was a strong increase in gene activation in the mid-exponential phase in the presence of cellobiose, glucose caused the highest percentage of overall gene expression activation (73% activation versus 27% reduction in chromosomal gene transcription).

**Figure 3:**
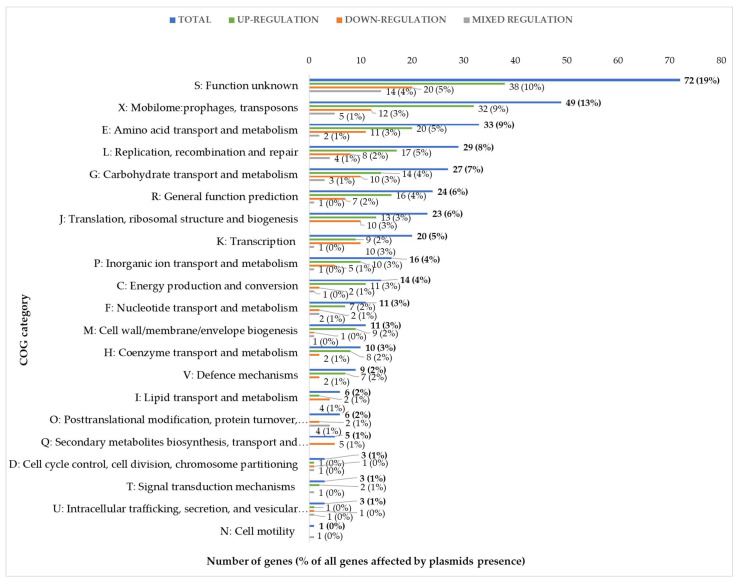
Numbers and percentage (in brackets) of unique differentially expressed chromosomal genes in *L. lactis* IL594 during growth on three carbon sources in four growth phases with identified COG categories. The percentages presented in brackets refer to the proportion of genes from the analysed COG category to the total number of genes with altered expression profiles.

The presence of plasmids alters the expression of chromosomal genes mainly with metabolic, defense, or regulatory functions. Because some of the genes differentially expressed in the presence of each of the three sugars overlapped (Supplementary Figure S2), we calculated that the total number of unique differentially expressed genes was 375, representing approximately 16% of all genes annotated in the *L. lactis* IL1403 genome. These 375 genes were grouped based on their biological role according to functional categories of COGs (Clusters of Orthologous Groups) (Figure 3). Genes encoding proteins of unknown function (COG category S) as well as prophage genes and transposons (COG category X) were the most strongly represented groups, with 71 and 49 unique genes, respectively, accounting for 32% of all identified changes in the IL594 transcriptome. More than 31% of the other differentially expressed genes (118 genes) encoded proteins with metabolic functions involved in seven COGs: Amino acid transport and metabolism (E, 33 genes), Carbohydrate transport and metabolism (G, 27 genes), Inorganic transport and metabolism (P, 17 genes), Energy production and conversion (C, 14 genes), Nucleotide transport and metabolism (F, 11 genes), Coenzymes transport and metabolism (H, 10 genes), and Lipid transport and metabolism (I, 6 genes). The third most numerous group with 71 genes (19%) regulated in the presence of plasmids were genes with regulatory functions involved in the processes of DNA replication, recombination and repair (L), transcription (K) and translation, ribosomal structure and biogenesis (J) (Figure 3).

The presence of plasmids in *L. lactis* IL594 led to significant changes in the expression of over 70 genes encoding proteins of unknown or hypothetical function (S, R), with activation of expression occurring more than twice as often as repression. Up-regulation of expression of 54 unique genes and down-regulation of 26, corresponding to 14% and 7%, respectively, of all genes regulated solely by the presence of plasmids (Figure 2). A positive effect of plasmids presence on the prophages genes expression (X) was also observed. Transcriptomic analysis showed significant up-regulation of genes from two *L. lactis* lytic phages (bIL286 and bIL310) and one temperate phage bIL310. In the presence of all seven plasmids, 16 genes from bIL286 and 17 genes from bIL310 (26% and 30% of all bIL286 and bIL310 phage genes, respectively) were upregulated during growth of *L. lactis* IL594 in medium containing cellobiose or galactose. Moreover, 16 bIL310 prophage genes (57% of all phage genes) were up-regulated exclusively in the plasmid-containing strain during growth under all culture conditions tested (Figure 2; Supplementary Table S2). Despite the induction of prophage gene expression, no significant cell lysis was observed in the presence of cellobiose or galactose.

Among genes with metabolic function, a large set of 33 chromosomal genes undergoing differential expression was assigned to amino acid transport and metabolism (category E) (Figure 2, Supplementary Table S2), for which a dominance of activation of expression was observed. The most significant 2.1-6.2-fold increase in gene expression at almost all growth stages for all carbon sources tested was observed in IL594 for the *oppBCDF* operon encoding two oligopeptide ABC transporters. Furthermore, in the context of substrate transport, several genes (*ydgB*, *yibG*, *yrfD*, *yshA*) encoding secondary transport systems (antiporters and uniporters) dedicated to amino acids uptake were up-regulated by 2.5-4.0-fold. In addition, the presence of plasmids elevated the expression of genes coding for amino acid synthetases (*asnH*, *cysK*, *gltB*, *ilvH*), dehydrogenase (*homD*), and phosphatase (*hisK*). Another set of genes encoded several peptidases with different specificity (endo-, dipeptidases) involved in casein degradation, which were activated (*pepD*, *pepA*, *pepF*) or inhibited (*pepO*, *pepP*) during the presence of IL594 plasmids. The strongest 2.3–3.1-fold transcriptional repression was observed for the *optAB* genes encoding oligopeptide ABC transporters, essential components of the di- and tripeptide transport system in *L. lactis*.

A further widely represented group of genes showing differential expression belonged to the COG category of carbohydrate transport and metabolism (G). In this category, several genes were identified whose expression was significantly inhibited in the presence of plasmids (Figure 2). Notable among these are the *malEFG* operon encoding a maltose-specific ABC transporter and *rbsDK* responsible for ribose degradation, whose expression was permanently down-regulated for all sugars, especially at the later growth stages. This group of down-regulated genes also included two mutases whose differential expression was detected at early growth stages on glucose or cellobiose (*pgmB*), or cellobiose only (*glmM*). For most of the other differentially expressed genes in the G category, a slightly more than twofold increase in their transcription was observed in IL594. These include genes for utilization of xylose or its derivatives, responsible for their transport (*xylT*, *xynT*) and subsequent conversion (*xylB*, *xynD*), as well as genes for galactose (*galE*), gluconate (*gntK*) and N-acetyl-glucosamine (*glmM*) metabolism. The presence of plasmids also weakly (up to three-fold) activated genes for phosphotransferase system (PTS)-driven uptake of carbohydrates such as fructose (*fruA*) and β-glucosides (*yedF*) as well as ABC transporters of polysaccharides (*yngG*, *ypdA*) (Supplementary Table S2).

Another set of differentially expressed genes included those from the COG category of energy production and conversion (C). In particular, a significant 2.1-8.1-fold up-regulation of genes involved in citrate (*citB*), lactate (*ldhB*) and malate (*mleS*) metabolism in IL594 was observed (Figure 2, Supplementary Table S2). Also in this category, the expression profile of genes related to redox reactions was significantly altered and included those up-regulated, such as several oxidoreductases (*ybiE*, *ycdG*, *yddB*, *ypjF*, *yrjB*) and two ATP synthase subunits (*atpCD*), and those with reduced expression such as *noxD*, *yfiJ*, and *yiaD* encoding NADH/NADPH oxidases and reductases.

The presence plasmids also resulted in changes in the expression level of 31 unique genes involved in the nucleotide (F), coenzyme (H) and lipid (I) transport and metabolism, representing 8% of all identified changes in the IL594 transcriptome (Figure 2). Among them, the up-regulation of seven genes and down-regulation of four other genes involved in nucleotide uptake and metabolism was observed in IL594. The most significant 2–3-fold increase concerned the expression of individual ribonucleases (*ygbG*, *ypdB*, *ypdD*) and genes involved in adenosine (*add*, *pfs*) and guanine (*xpt*) conversions. The transcription of ten genes involved in aerobic and anaerobic cell metabolism throughout coenzyme transport and metabolism was also observed with the significant prevalence of expression up-regulation (eight genes). For most of the other differentially expressed genes in the “H” category, slightly more than a two-fold increase in their transcription was observed in IL594. The genes encoding genes involved in aerobic respiratory metabolism (*menD*) and anaerobic electron transport (*menB*, *ribA*) as well as pyrimidine and NAD biosynthetic pathways (*nadD*, *pyrZ*) were upregulated in the presence of plasmids. Conversely, the *difpA* gene of coenzyme A biosynthesis pathway and the *ychG* gene involved in heme synthesis and transport were twofold repressed in IL594. Six genes of lipid transport metabolism were also differentially regulated by the presence of plasmids. Genes encoding enzymes of lipid metabolism, including acetyl-CoA carboxylase (*accD*), hydroxymethylglutaryl-CoA reductase (*mvaA*) and (3R)-hydroxymyristoyl-ACP dehydratase (*fabZ1*) were most noticeably downregulated by 3-fold during early-exponential and mid-exponential growth phase in IL594.

Among genes from the COG categories Inorganic ion transport and metabolism (P) and Defence mechanisms (V), IL594 strain had alleviated expression of 17 genes, corresponding to 5% of all genes regulated exclusively by plasmids presence. The up-regulation of genes involved mainly in bivalent cation transport such as magnesium (*pacL*, *yoaB*) or heavy metals (*cbiO*, *ydaE*, *yogJ*, *yuiA*, *zitQ*) were observed mostly in early-exponential and mid-exponential growth phase in IL594 strain. Conversely, the expression of *mtsABC* gene cluster encoding components of manganese import pathway and two genes encoding ferrum (*fhuB*) and magnesium (*ygfE*) importers were negatively regulated. In plasmid-containing *L. lactis* IL594 strain the activation of genes involved in multidrug resistance was also observable. The multidrug resistant efflux pumps (*lmrAP*, *ycfC*, *ydaG*, *ypbC*, *yvhA*, *ywiG*) were noticeably up-regulated in the presence of plasmids. The *lmrAP* encodes a proton/drug antiporter (LmrP) and an ATP-dependent primary transporter (LmrA). The *ydaG* gene encoding ATP-binding cassette half-transporter and the gene encoding kanamycin kinase (*ymdC*) were both up-regulated. On the other hand, in the presence of plasmids in *L. lactis* IL594 the expression of genes encoding type I restriction enzyme (*hsdS*) and permease component of ABC-type multidrug transport system (*ypgD*) were negatively affected.

The effect of plasmids presence also affected the expression of regulatory genes and resulted in significant variation in the expression of 20 genes listed in the COG category K. Plasmid-induced activation as much as repression of genes encoding transcriptional regulators was observed in this group. Among them, a bunch of genes encoding hypothetical regulators of the TerR/AcrR family (*ybeD*, *ycfA*, *yohC* and *ygfC*) or GntR family (*rgrA*) was strongly inhibited, as well as regulatory genes with defined function such as *mleR* and *rbsR* encoding, respectively, malolactic fermentation activator and the repressor of the ribose metabolism operon. On the other hand, the activation of expression was observed for the *pyrR* gene encoding the pyrimidine biosynthesis regulatory protein as well as several genes with putative regulatory function (e.g., *ynaB*, *rliB*, *rmaE*, *yjaJH*) belonging to different families such as LacI, MarR, or LytR. 

In the COG functional category Replication, recombination and repair (L), a total of 29 genes were differentially expressed in the presence of plasmids in *L. lactis* IL594. Significant part of the activated ones included a wide group of transposons, transposases and a hypothetical group of transposon-related function (15 genes). The other genes with elevated expression profiles included these coding for DNA polymerase III (*polC*), DNA glycosylase (*mutM*), exonucleases (*xseA*, *sbcC*), and DNA repair protein (*recO*). Conversely, the genes exclusively repressed in the presence of plasmids were encoded components of the DNA polymerase III system (*dnaN*, *dnaQ*), primosomal protein DnaI (*dnaI*) and ssDNA-specific exonuclease (*recJ*).

In other COG categories such as Translation, ribosomal structure and biogenesis (J) and Cell wall/membrane/envelope biogenesis (M), in total 33 unique genes were expressed differentially in plasmid-harboring strain, representing 9% of all identified changes in the IL594 transcriptome. The significant up-regulation of 12 genes and down-regulation of 10 others involved in translation processes was observed in IL594, most of which represented genes encoding 30S and 50S ribosomal proteins as well as some tRNA synthetases (Supplementary Table S2). For almost all of the differentially expressed genes in the “M” category, slightly more than a two-fold increase in their transcription was observed in IL594. The genes encoding proteins involved in peptidoglycan and LPS biosynthesis (*dacB*, *glmS*, *murB*, *rgpE*, *ycbF*, *ycbG*) as well as components of outer membrane (*plpB*) and antibiotics binding proteins (*pbp2A*) were upregulated in the presence of plasmids.

## 3. Discussion

The studies described in this work demonstrate the phenotypic and transcriptomic differences between the industrially important multiplasmid *L. lactis* strain IL594 and its plasmid-cured derivative *L. lactis* IL1403. It was previously thought that the presence of seven plasmids could lead to adaptive and industrially important traits in this strain [7]. Here we confirm these observations through phenotypic and transcriptomic studies, as the presence of plasmids caused a change in the expression of about 16% of chromosomal genes and in the rate of cell growth, an improved or even emerged ability to assimilate new carbon sources, enhanced cell resistance to some toxic metal ions, antibiotics, and other antimicrobial agents. However, it is important to note that not all observed changes in phenotypic traits are due to plasmid-chromosome cross-talk, but can also be the result of the direct presence and activity of plasmid genetic load, which we will attempt to delineate below.

The first observed feature of the multiplasmid strain was its slightly delayed growth rate compared to the plasmid-free strain, both under repressive (glucose) and non-repressive cultivation conditions (cellobiose), indicating the influence of plasmids on bacterial fitness. Plasmids usually cause a metabolic burden on the host, which was also reported in previous studies concerning lactococcal plasmids [8]. The accepted explanation for this phenomenon is that plasmid-containing cells require more energy and nutrients for replication and synthesis of plasmid gene products than the plasmid-free cells. It was suggested that plasmids compete with the chromosomally encoded genes for limited cellular reserves for DNA replication and expression. As a result, plasmids can affect host cells traits such as growth rate and yield constancy [9]. On the other hand, the growth rate of *L. lactis* IL594 in galactose- supplemented medium or its metabolic activity on lactose and its derivative (lactulose) as well as derivatives of galactose (3-O-β-D-galactopyranosyl-D-arabinose and 4-O-β-D-galactopyranosyl-D-glucitol [D-lactitol]) were apparently enhanced or enabled at all. This trait may be due to the direct activity of plasmid genes, as the pIL4 plasmid contains genes encoding the complete lactose-specific phosphotransferase system (*lac*-PTS) and the components of the tagatose-6-phosphate enzymatic pathway, that may be responsible for the more efficient galactose and lactose fermenting abilities [10]. This observation stays in line with previous reports, which indicate that rapid lactose fermentation represents mostly plasmid-encoded ability of LAB strains acquired by wild-type plant strains as a result of adaptation to a milk environment [11]. However, plasmid-free IL1403 was still able to utilize galactose and lactose, which may be explained by the presence of the chromosomal Leloir system for efficient galactose metabolism [12] and a cellobiose-specific PTS system that enables slow lactose utilisation after prolonged incubation [13,14]. In addition, the rapid conversion of lactose to lactate results in acidification of the cytoplasm with effects on membrane potential. The presence of plasmidic *citP* and *oxlT* genes encoding pH gradient equalizing proteins in IL594 may also lead to fast and efficient utilization of lactose [7]. However, verification of this hypothesis requires further functional analyses.

We have not observed a significant effect of plasmids on the metabolism of other carbon sources even using such an advanced and extensive technique as phenotypic microarrays. This small number and intensity of changes caused by the presence of plasmids may be due to the quite wide range of carbon source metabolism by *L. lactis* IL1403 when compared with other plasmid-free lactococci [15]. Among these a few remaining carbon sources whose metabolism was enhanced in the plasmid strain, two β-glucosides, gentiobiose and esculin, were prominent, and the third, arbutin, was also noticeable, but to a lesser degree. No similar effect was observed for other β-glucosides, since both strains utilized cellobiose and salicin in a similar manner. In β-glucosides such as cellobiose and gentiobiose, the glucose moiety is linked to a second glucose residue, while in arbutin, esculin and salicin, it is joined to a non-sugar residue. In the cytosol, imported by β-glucoside-specific PTSs, phosphorylated β-glucosides are hydrolyzed by various P-β glucosidases into simpler compounds, which further enter different metabolic pathways depending on their chemical nature. The analysis of the chromosomal sequence of *L. lactis* IL1403 indicates the presence of genes encoding homologs of various beta-glucoside-specific PTS components (three-domain IIABC PTS components [PtbA, YedF, YleE], IIC permeases [CelB, PtcC, YidB], IIA and IIB components [PtcA and PtcB]), and at least six P-β-glucosidases (BglA, BglH, BglS, YidC, YpcA, YrcA). While the pathway for cellobiose transport and hydrolysis in *L. lactis* IL1403 has already been elucidated and attributed to CelB, PtcA, PtcB and BglS [13], the genes as well as the mechanisms involved in the assimilation of the remaining β-glucosides are not yet known in this strain. In light of this lack of knowledge regarding β-glucoside metabolism, it seems interesting to find here the differential expression of genes encoding YedF and BglH, which were activated in the presence of plasmids in IL594 and therefore may be responsible for the increased metabolism of gentiobiose, esculin, and arbutin in this strain. The activation occurred under most of the conditions tested, both in the presence of monosaccharides (glucose and galactose) or cellobiose, suggesting a constitute induction of gene expression caused by the presence of plasmids, and most likely by the influence of some regulatory elements carried by them. Such control can be direct through the mere action of a plasmid regulatory protein on a chromosomal structural gene, or indirectly through a cascade of regulation involving the influence of plasmid regulators on chromosomal ones, which in turn affect other genes. Indeed, in support of the latter supposition, changes in the expression of a number of chromosomal regulatory genes have been identified in the presence of plasmids in IL594, many of which have yet to be assigned a function. In addition to *yedF*, some other carbohydrate transport genes encoding primary and secondary transporters (ABC transporters and porters, respectively) as well as PTS are also overexpressed in the presence of plasmids. Among them, the genes responsible for the transport and/or subsequent metabolism of xylan, xyloside and xylose stand out in particular. Interestingly, *L. lactis* IL594 and IL1403 were unable to grow in the presence of xylose in both the D and L configurations. It is assumed that dairy lactococal strains, unlike those of plant origin, do not have adequate systems to metabolize this carbohydrate [16]. However, *L lactis* IL1403, despite its dairy origin, carries a whole system dedicated to xylose metabolism, but probably one which is nonfunctional due to the C-terminal truncation of the D-xylose proton symporter, encoded by *xylT* [17]. The observation of increased expression of the *xyl* genes in *L. lactis* IL594 suggests that in Xyl^+^ strains, the presence of similar plasmids could further enhance the metabolism of xylose and its derivatives.

Resistance to metal ions, antibiotics and other antimicrobial agents play a key role in the adaptation of LAB strains to dynamically changing environmental conditions and often determines the potential for industrial exploitation of the strains. When profiled using chemical sensitivity phenotype microarrays, IL594 and IL1403 were tolerant to a fairly wide range of antibiotics, such as ampicillin, moxolactam, piperacillin, geneticin, cefoxitin, cefmetazole, phleomycin, nitrofurantoin, nitrofurazone, furaltadone, oxolinic acid, or chloramphenicol, as well as to several toxic ions, particularly cations such as boron, and selected transition or alkali metals. The identified features of antibiotic resistance are a phenomenon previously observed among LAB strains, including those isolated from pharmaceutical products [18], probiotic starter cultures [19], and traditional fermented food [20]. The presence of *Lactobacillus* or *Lactococcus* strains resistant to streptomycin, vancomycin, tetracyclines, erythromycin, ciprofloxacin or gentamicin was previously identified in pharmaceutical and dairy products [21,22,23]. The highly conserved antibiotic resistance in LAB strains is based on chromosomally encoded multidrug resistant (MDR) efflux pumps involved in the expulsion of a wide range of structurally unrelated substrates [19]. At least a dozen drug-related genes encoding putative MDR efflux pumps are present in the chromosome of *L. lactis* IL1403 [24]. Here, transcriptomic analysis revealed the effect of plasmids on increasing the expression of eight chromosomal genes that may be involved in enhanced resistance to toxic compounds, including *ymdC* coding for an aminoglycosides resistance protein and seven genes for different types of MDR efflux pumps. Among the latter are the *lmrA*, *ycfC*, *ydaG* and *ywiG* genes encoding multidrug resistance transporters of the ABC superfamily, *lmrP* encoding a transporter of the major facilitator superfamily (MFS), and *ypbC* and *yvhA* encoding transporters of the multidrug and toxic compound extrusion (MATE) family. Of these genes, the functionality of *lmrP*, *lmrA* and *ydaG* has been confirmed in previous studies in *L. lactis* as being involved in conferring resistance to various cytotoxic compounds and many clinically relevant antibiotics, including tetracyclines, macrolides, streptogramins and aminoglycosides [25,26]. Although in the vast majority of cases, the presence of plasmids had no strong effect on the antibiotic/drug resistance of *L. lactis* IL594, the modest increases in tolerance to some of these compounds may be correlated with the activation of the referred genes for MDR efflux pumps. However, this supposition needs to be confirmed in further studies. Similarly, some LAB strains have previously shown resistance to toxic ions through efflux transporters [27]. In this study, of the 17 toxic ions tested, the presence of plasmids generated a general but a relatively small increase in the tolerance of strain IL594 to many of them. However, several of the observed ion resistance features of the *L. lactis* IL594 strain can be directly linked to the presence of plasmidic genes. The genes encoding the Cd^2+^/Ni^2+^ ion transport system (*cad* operon) are located in the pIL5 plasmid, which may indicate a key role of the this plasmid in determining the resistance of IL594 strain to metalloids. The two-component operon consists of the regulatory *cadX* and resistance *cadD* genes, encoding resistance of Cd^2+^, Co^2+^ and Zn^2+^ efflux systems [28]. In that plasmid, several genes encoding putative transporters may also be involved in the transport of inorganic ions (e.g., the *orf16* gene encoding putative Mn^2+^/Fe^2+^ ion transporter) [7]. However, better adaptation to the presence of toxic ions may also be due to the effect of plasmids on chromosomal gene activity. This assumption is supported by plasmid-induced changes in the expression of a number of chromosomal genes from the Inorganic ion transport category, in particular, the activation of the transcription of *ydaE* and *yogJ* encoding components of potential efflux systems involved in the ejection of bivalent heavy metal ions of elements such as Cd, Zn and Co. In addition, an intriguing phenomenon observed here is the plasmid-induced repression of all three genes of the *mtsABC* operon encoding components of the manganese ABC transporter, whose function has already been fairly well described regarding manganese ion import in bacteria, and in particular in pathogenic streptococci [29]. Manganese is an important transition metal used by organisms as an essential trace element, but it can also be potentially harmful, requiring accurate regulation of its homeostasis [30]. Thus, we can assume that the presence of plasmids, by reducing the activity of the MtsABC system, could protect the cell from an excess supply of manganese, and thus in their presence the bacterium will fare better in an environment of excess ions of this metal. Indeed, as a confirmation of this hypothesis, we observed in this study an increase in tolerance to manganese ions in the plasmid-carrying strain IL594 compared to its plasmid-free derivative.

The presence of plasmids leads to an increase in host adaptive functions, and in the case of this study, this was a clear and broad, albeit not drastic, trend at both the genotypic and phenotypic levels. The aforementioned characteristics, which can provide better adaptation to different environmental conditions, are also important for modulating biotechnological fermentation processes. Further studies should attribute changes in phenotypic variation and in chromosomal gene expression to specific plasmids or even genes.

## 4. Materials and Methods

Bacterial strains and growth conditions. *L. lactis* strains used in this study are shown in Supplementary Table S1. Bacteria were grown aerobically at 30 °C in M17 (Oxoid Ltd., Basingstoke, Hampshire, UK) supplemented with 1% (*wt*/*vol*) glucose (M17-Glu), galactose (M17-Gal) or cellobiose (M17-Cel). Solidified media contained 1.5% agar (Sigma-Aldrich, St. Louis, MO, USA).

Plasmid copy number identification. Plasmid cell copy number in *L. lactis* IL594 was determined using quantitative polymerase chain reaction (qPCR) technique. Total RNA was isolated using GeneMATRIX Universal RNA Purification Kit (EURx, Poland) from 10 mL of *L. lactis* IL594 at mid-exponential phase (OD_600_ = 0.7) for three independent cultures grown in M17-Cel. RNA was treated with DNAse I (Sigma-Aldrich, USA), and its quality and quantity were checked by agarose gel visualization and Nano-drop spectrophotometer (Thermo Scientific, Waltham, MA, USA). First-strand cDNA was obtained using the RevertAid (TM) First-Strand cDNA Synthesis Kit (Thermo Fisher Scientific) with random primers according to manufacturer’s instructions. The chromosomal genes *purM* and *tufA* were selected as references for the qPCR reaction. For each plasmid sequence, two specific plasmid genes (a total of 14 plasmid-localized genes) without chromosomal duplicates were amplified in the qPCR reaction. Real-time quantitative PCR assays were performed on a PikoReal 96 Real-Time PCR System instrument (Thermo Scientific, USA). Primers (Supplementary Table S1) were designed in Primer Quest software (http://eu.idtdna.com/PrimerQuest/Home/Index, accessed on 6 January 2022). Each reaction was carried out in 10 μL of reaction mixture containing: 1× concentrated SYBR Green RT PCR MIX SYBR^®^ A (A&A Biotechnology, Gdańsk, Poland), specific forward and reverse primers (100 μM each), cDNA template (in three amounts per well—9, 3 and 1 ng, each in duplicate). Reactions were performed with an initial denaturation step (95 °C; 1 min) followed by 50 cycles of denaturation (95 °C; 12 s) and primer annealing-extension (60 °C; 30 s). Fluorescence was read during the annealing-extension step of each cycle. After the cycle, melting point temperature analysis was performed from 60 °C to 95 °C with a temperature increment of 0.1 °C. The quality of the results was evaluated based on the expected Ct differences between the three cDNA amounts the melting curves of the products. The three cDNA concentrations allowed calculations of individual yields for each primer pair and normalization of all results to a single cDNA concentration common to all genes. Rare outlier results were omitted form the calculations. The amount of each target gene was calculated using a modified ΔCt method, taking the geometric mean of two common Ct values as reference [10].

Metabolic activity and sensitivity assays. Metabolic profiles were measured globally using the Phenotype MicroArrays (PM) system (Biolog Inc., USA), according to the manufacturer’s instructions. Briefly, the method involves detecting metabolic activity through the chemistry of tetrazolium dye. NADH formed in cellular metabolism reduces colorless tetrazolium dye to purple formazan. Based on cellular metabolism, the development of color over time is arranged in a sigmoidal curve of metabolic activity, similar to a traditional growth curve. *L. lactis* strains were cultured on M17-Glu solid medium, and the grown colonies were titrated in IF-0a inoculating fluid until the solution reached the desired transmittance of 81%. Growth supplements and tetrazolium redox dye D (Biolog Inc., USA) were added according to standard protocols recommended by Biolog Inc. for *Streptococcus* spp. 100 μL aliquots were added to each well in PM panels and incubated in an OmniLog incubator-reader (30 °C; 96 h). Colorimetric data of tetrazolium dye reduction kinetics were collected approximately every 15 min and analyzed using OmniLog PM software (Biolog Inc., USA). The manufacturer does not specify substrate concentrations in the wells, and the expression of color formation is recorded in arbitrary Omnilog units (OAUs). For the purpose of this work, the areas under the curves (AUC) of metabolic activity of the strains were calculated, averaged and presented in OAUs.

Oligonucleotide microarrays. *L. lactis* IL594 and IL1403 cells used for RNA isolation were obtained from four distinct growth phases: early exponential [E] at OD_600_ 0.1–0.2, mid-exponential [M] at OD_600_ 0.6–0.7, transition [phase between the exponential and stationary growth; T] at OD_600_ 0.8–0.9, and stationary [S] after 12 h of incubation (Figure S1). The total RNA was isolated from 10 mL of *L. lactis* cultures grown in M17 medium supplemented with desired sugar (cellobiose, galactose or glucose) using a Gene MATRIX Universal RNA Purification Kit (EURx, Danzig, Poland), according to the manufacturer protocol. Total RNA quality was checked by visualization on agarose gel, and the concentration and purity were determined with a Nano-drop spectrophotometer (Thermo Scientific, USA). Cy3 or Cy5 fluorescently labeled cRNA were synthesized with 100 ng total RNA as template using the Agilent Quick Amp Two-Color Labeling Kit (Agilent Technologies, Santa Clara, CA, USA) according to the manufacturer’s protocol and obtained cRNA was quantified using a NanoDrop spectrophotometer. Comparative microarray hybridizations of IL594 and IL1403 transcriptomes were performed in three biological replicates from cRNA probes obtained from four distinct growth phases during cultivation on glucose as well as from cRNA probes obtained from mid-exponential and transition growth phases during cultivation on cellobiose. Other hybridizations were performed in one biological replicate. All of the hybridizations were performed with two technical replicates with dye-swap. Custom Gene Expression 8 × 15 K Microarray slides (Design ID: 084868) containing 2441 60-mer oligonucleotide probes representing all known *L. lactis* IL594 genes were used (Agilent Technologies, USA). The resulting fluorescence images were scanned with the Axon GenePix 4000B (Molecular Devices, San Jose, CA, USA) microarray scanner. Feature extraction was accomplished with GenePix Pro 6.1. The raw data were Lowess-normalized, averaged, and statistically analyzed using Acuity 4.0 software (Molecular Devices). For each gene, a LogRatio value expressing over- or underrepresentation of its transcript in compared samples was calculated. A gene was considered differentially expressed between compared *L. lactis* strains in given growth phase and the carbon source if the level of its transcript differed by at least 4-fold (|Log2Ratio| ≥ 2.0) with the statistical significance, *p* < 0.05. The resulting lists of differentially expressed genes (DEGs) were further subjected to bioinformatic analysis. ORF annotations of *L. lactis* were retrieved from the BASys Omix software [9]. Genes with altered expression profiles were grouped based on putative functions of the encoded proteins into COG categories using the WebMGA RPS-blast tool [31]. The complete transcriptome analysis data were deposited in the Gene Expression Omnibus (https://www.ncbi.nlm.nih.gov/geo/, accessed on 26 July 2022) under accession no: GSE209756.

## Figures and Tables

**Table 1:** Numbers of all genes whose expression profile was altered by the presence of pIL1-7 plasmids from *L. lactis* IL594 during growth on different sugars and at different growth phases. ↑, number of up-regulated genes; ↓, number of down-regulated genes; ∑, sum of up- and down-regulated genes per growth phase.

	Number of Genes Affected												
Carbon Source	Early Exponential Phase			Mid- Exponential Phase			Transition Phase			Stationary Phase			Total Changes Identified
	∑	↑	↓	∑	↑	↓	∑	↑	↓	∑	↑	↓	
Cellobiose	93	59	34	157	129	28	81	21	60	51	29	22	382
Galactose	105	50	55	42	21	21	70	49	21	15	11	4	232
Glucose	21	12	9	31	17	14	26	21	5	51	45	6	129

## Data Availability

Transcriptome analysis data are available from the Gene Expression Omni-bus (https://www.ncbi.nlm.nih.gov/geo/, accessed on 11 July 2022) under accession no: GSE209756.

## Supplementary Materials

The following supporting information can be downloaded at: https://www.mdpi.com/article/10.3390/ijms24010793/s1.
