# Supplementary material for: The Presence of Plasmids in Lactococcus lactis IL594 Determines Changes in the Host Phenotype and Expression of Chromosomal Genes
Source: Int J Mol Sci. 2023 Jan 2;24(1):793. doi: 10.3390/ijms24010793 (PMC9821262; doi:10.3390/ijms24010793)
Supplement: Supplementary file 1 [file ijms-24-00793-s001.zip › ijms-2065741-supplementary.pdf]

The presence of plasmids in *Lactococcus lactis* IL594 determines changes in the host phenotype and expression of chromosomal genes

Katarzyna Kosiorek[1], Anna Koryszewska-Bagińska[2], Marek Skoneczny[1], Lidia Stasiak-Różańska[3] and Tamara Aleksandrzak-Piekarczyk[1,*]

[1] Institute of Biochemistry and Biosciences, Pawinskiego 5a, 02-106 Warsaw, Poland
[2] Department of Medical Biology, Medical University of Warsaw, Litewska 14/16, 00-575 Warsaw, Poland
[3] Department of Food Technology and Assessment, Institute of Food Sciences, Warsaw University of Life Sciences-SGGW, Nowoursynowska 159C St., 02-787 Warsaw, Poland
* Correspondence: tamara@ibb.waw.pl; +48 592 12 13

Supplementary Materials

Table S1. Strains and primers used in this study.

Strain or primer pair	Genotypic characteristics	Source and reference
L. lactis		
IL594	wild-type strain harbouring seven plasmids pIL1-pIL7	INRA [31]
IL1403	plasmid-free strain, derivative of IL594	
**Primers[a]**		
LlPurMaF / LlPurMaR	ATTGCGTAGCCATGTGCGTC/CTGTTTCTCCACCAATCAGCG	This study
LlTufaF / LlTufaR	CGTGACCTCTTGAGCGAATACG/GAGTGGTTTGTCAGTGTCGCG	
p1orf3zF / p1orf3zR	CTTGCTGACGAATTAGGAGTTAG/TCAGCGGTTAATAGCTTCATAC	
p1orf6zF / p1orf6zR	CCTTGTTCGTTGGATTGATTAG/AGAACTCGCTTCTAAATTTGTG	
p2citRzF / p2citRzR	AGGAGACAACAACATGAAAGTC/GAGCGTTTGTCTCCAATCTG	
p2orf2zF / p2orf2zR	AGCTAAACAGACCGTTCAATAC/GTTTAGGACGATCGAACCAC	
p3orf2tF / p3orf2tR	CAACAACAGAACGAAACACAAC/AAGTTCTGTTCTAAGAATACTGACG	
p3orf3zF / p3orf3zR	TCTCAGGTGTTTATCTGGTATTTC/GACAACAAGTCCTACTCCAAAG	
p4or24zF / p4or24zR	GTTTGATCTTACCTTGCTGTTG/GTTACTGGGAACCGATTACG	
p4or49zF / p4or49zR	CCTTACCAATGGTGATGTAATCG/GTCAAAGATACTGGCGGTTTAG	
p5cadAzF / p5cadAzR	GCATCGACGGTTGGTATTG/GTTTGGCGGCTAAGTCTAAC	
p5cadCzF / p5cadCzR	GTTGATAGTCACAAGGATGGTAAG/AATTTACGCCAAGCTCCATAAG	
p6orf6zF / p6orf6zR	TCAGGGAATTCTTTATTAGGTTCTG/CTTGTTCATCTACCTTAGTCAAGTATC	
p6orf7zF / p6orf7zR	GATATACTGCGACTTCCAGTTG/ATTCTAATGCCACATGGTAAGG	
p7or11zF / p7or11zR	ATCGAACCGCCAATCAAG/CCGTATTGATCCCAGAAGTAAG	
p7orfXzF /p 7orfXzR	TGCAGATGAGTTGAGTGTTAC/TGCTACCACTTTCTACTTTACC	

INRA - French National Institute for Agricultural Research
[a]primers were designed on the basis of the *L. lactis* IL594 plasmid nucleotide sequences (NCBI accession no. HM021326, HM021327, HM021328, HM021329, HM021330, HM021331, and HM197723; http://www.ncbi.nlm.nih.gov)

Figure S1. Time points of samples collection during growth of *L. lactis* IL594 (---) and IL1403 (•••) in M17 supplemented with different carbon sources. Identified time points of bacterial growth phases: early exponential (E), mid-exponential (M), transition (T), and stationary (S) are presented in circles.

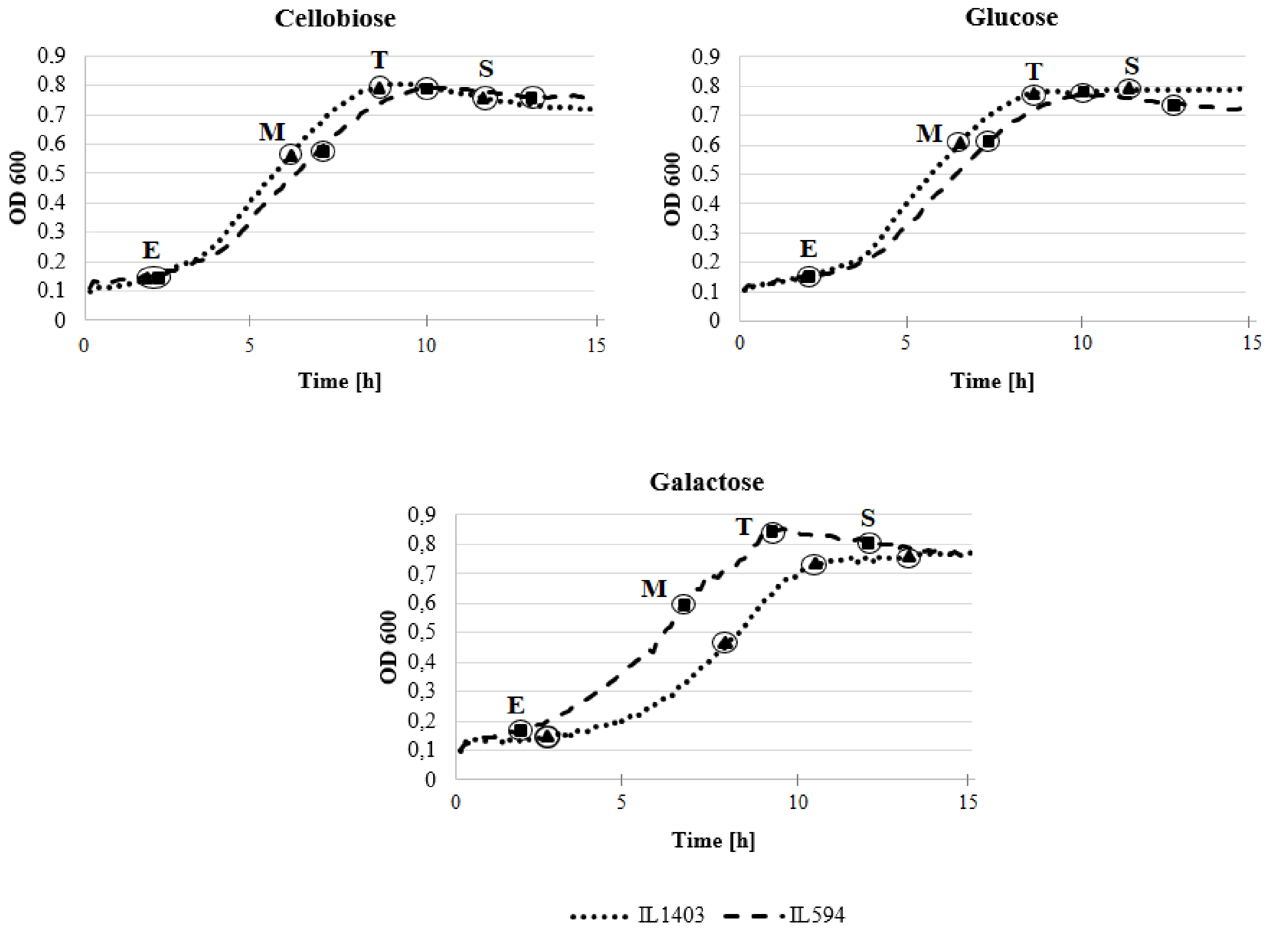

Figure S2. Venn diagram of differentially expressed genes in *L. lactis* IL594 in comparison to IL1403 induced by presence of different carbon sources (glucose- “Glu”; cellobiose- “Cel”; galactose- “Gal”). The numbers of genes in the intersection sets are indicated. E refers to the early exponential phase of growth, M - mid-exponential growth phase, T - transition phase and S - stationary phase of growth. Overlapping regions show the number of common genes with an altered expression profile at different growth phases. Numbers in non-overlapping areas of each oval show the number of genes expressed differently only in selected phase of growth unique to each strain. The number of genes expressed commonly in all growth phases included 7 (0.3%), 8 (0.3%), and 2 (0.08%) genes during growth of the strain on glucose, cellobiose, and galactose, respectively. The number of genes expressed differently in all growth phases included 49 (2%), 146 (6%), and 140 (5%) genes during growth of the strain on glucose, cellobiose, and galactose, respectively. The numbers in brackets represents the percentages of all genes annotated in IL1403 genome.

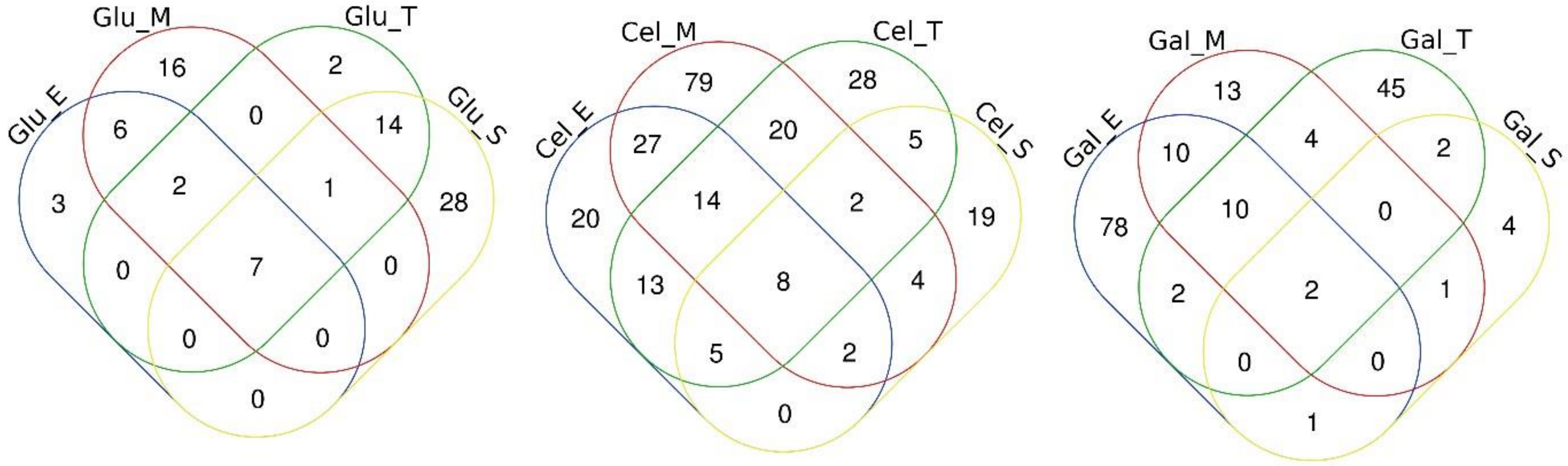

Table S2 . Differentially expressed genes in *L. lactis* IL594. A gene was considered to be upregulated between compared L. lactis samples if the level of its transcript was elevated by at least 4-fold (Log2Ratio≥2.0) and downregulated when the level of its transcript was decreased by at least 4-fold (Log2Ratio≤-2.0). The transcription change was recognized as valid when the probability of such a random difference was less than 0.05 ($p < 0.05$).

Gene	NCBI GeneID	COGs	Upregulation or downregulation on a sugar source												Protein function
			Glucose				Cellobiose				Galactose				
			E	M	T	S	E	M	T	S	E	M	T	S	
**Energy production and conversion**															
atpC	SPS12515.1	C		2.6				2.8			2.4				F0F1 ATP synthase subunit epsilon
atpD	SPS12514.1	C					2.3		2.5				3.5		F0F1 ATP synthase subunit beta
citB	AAK04769.1	C				2.9	5.5	6.3	7.5	3.8	7.8	8.1	6.0		aconitate hydratase
ldhB	AAK04474.1	C					2.4	3.0							L-lactate dehydrogenase
mleS	AAK04998.1	C			2.1					2.9			2.3	2.1	malate dehydrogenase malic enzyme
noxD	GEB09238.1	C						-4.1	-2.0		-3.6	-4.2			NADH oxidase
ybiE	GFO78088.1	C	2.3					3.3			2.2				oxidoreductase
ycdG	AAK04334.1	C								2.2	4.9				oxidoreductase
yddB	AAK04431.1	C				2.2									oxidoreductase
yfhC	AAK04676.1	C				2.0				2.1					hypothetical protein
yfiJ	AAK04695.1	C	-2.0						-2.5	-2.1	-2.3				NADPH-flavin oxidoreductase
yiaD	AAK04894.1	C						-2.5	-2.0						NADH-flavin reductase
ypjF	AAK05652.1	C						2.7							oxidoreductase
yrjB	AAK05831.1	C		2.1					-2.3						oxidoreductase
**Cell cycle control, cell division, chromosome partitioning**															
ftsE	SPS11645.1	D					-2.2	-2.2				2.3			cell-division ATP-binding protein
ftsW2	AAK04982.1	D				2.5							3.1		hypothetical protein
gidA	AAK05945.1	D						-2.3							tRNA uridine 5-carboxymethylaminomethyl modification protein GidA
**Amino acid transport and metabolism**															

arcA	WP_004254504.1	E									-2.9				arginine deiminase
arcC2	AAK06131.1	E				-2.7									carbamate kinase
aroD	SPS10137.1	E									-2.3				3-dehydroquinate dehydratase
asnH	AAK06326.1	E						2.6	2.0						asparagine synthetase
cysD	AAK04171.1	E											2.5		O-acetylhomoserine sulfhydrylase
cysK	QOK50869.1	E					-3.5					4.2	-2.3		cysteine synthase serine family
glnB	AAK05689.1	E		3.0			2.5	3.1			2.4				nitrogen regulatory protein P-II
glnQ	SPS12229.1	E						-2.4	-2.4						glutamine ABC transporter ATP-binding protein
gltB	SPS11857.1	E						2.3							glutamate synthase large subunit glutamate family
hisK	GEB08549.1	E									2.4				histidinol phosphatase histidine family
homD	CAA65713.1	E										3.7	2.2		homoserine dehydrogenase
ilvD	SPS12837.1	E				2.2				2.2					dihydroxy-acid dehydratase
ilvH	SPS12840.1	E						2.0	-2.1						acetolactate synthase 3 regulatory subunit
oppB	QQF01347.1	E	6.0	6.7	6.3	6.1	6.4	6.8	8.1	3.0	7.0	6.7	5.4	0.8	oligopeptide ABC transporter permease
oppC	QQF01348.1	E	6.2	6.8	6.7	4.9	3.2	5.4	4.9		5.9	5.6	2.7	2.3	oligopeptide ABC transporter permease
oppD	QQF01345.1	E	4.2	4.9	3.3		3.2	5.4	4.9		5.9	5.6	2.7	2.2	oligopeptide ABC transporter ATP binding protein
oppF	QQE99754.1	E	5.0	6.0	5.0	4.0	4.4	6.7	6.4	2.3	6.2	6.2	3.4		oligopeptide ABC transporter ATP binding protein
optA	GEB08281.1	E									-3.1				oligopeptide ABC transporter
optB	GEB08280.1	E									-2.6	-2.3			oligopeptide ABC transporter permease
pepD	AAK04350.1	E					2.8	4.5							dipeptidase
pepF	CAA83534.1	E			2.7	2.1									oligoendopeptidase F
pepO	QOK51376.1	E					-3.9	-3.9			-2.8	-3.3	-2.5		neutral endopeptidase
pepP	AAK04789.1	E					-2.9		-3.0				-2.1		aminopeptidase P
trpC	TRW54532.1	E									-2.0				Indole-3-glycerol phosphate synthase
ydgB	AAK04453.1	E						2.8							L-asparagine uniporter

yejJ	AAK04598.1	E										2.0	hypothetical protein
yfcG	AAK04637.1	E										3.2	oligopeptide ABC transporter ATP binding protein
ygaJ	AE005176.1	E						-2.2					putative peptidase
yhbF	AAK04808.1	E					-2.4						hypothetical protein
yibG	AAK04907.1	E					2.6						L-asparagine uniporter
yrfD	AAK05797.1	E				2.7	4.0						amino acid antiporter
yshA	AAK05907.1	E					2.5						glutamate antiporter
**Nucleotide transport and metabolism**													
add	AAK04385.1	F					2.2	2.0					adenosine deaminase
dukA	GEB08937.1	F								2.4			deoxynucleoside kinase nucleotide and nucleoside interconversions
pfs	AAK05978.1	F					2.0						5'-methylthioadenosine/S-adenosylhomocysteine nucleosidase
xpt	AAK05244.1	F			2.9								xanthine phosphoribosyltransferase
yccE	AAK04320.1	F								-2.1			hypothetical protein ǀ NUDIX hydrolase
yejD	AAK04591.1	F								-3.4			hypothetical protein ǀ nucleoside 2-deoxyribosyltransferase
yfcD	AAK04634.1	F					2.7						hypothetical protein
ygbG	AAK04728.1	F					2.8						ribonuclease Z
ygiJ	AAK04786.1	F					2.5	-2.1					hypothetical protein ǀ adenylate kinase
ypdB		F				2.0					2.3		ribonucelase
ypdD		F				2.1				2.1			ribonucelase
**Carbohydrate transport and metabolism**													
bglH	AAK05557.1	G	2.0										beta-glucosidase
deoB	AE005176.1	G						-2.4					phosphopentomutase
dexC	GFO79576.1	G								2.5			neopullulanase degradation of polysaccharides

fruA	AAK05057.1	G		2.3											PTS system fructose-specific transporter subunit IIBC
galE	WP_058218180.1	G						2.2							UDP-glucose 4-epimerase
glmM	SPS12287.1	G						-2.0							phosphoglucosamine mutase
gntK	QNL92507.1	G						3.1	5.3	2.9	5.8	4.8	3.6		gluconate kinase energy metabolism, sugars
malE	AAK05781.1	G	-6.1	-5.8	-5.1	-3.1	-6.7	-6.3	-4.9	-2.0		-2.7	-3.0	-3.3	maltose ABC transporter substrate binding protein
malF	AAK05782.1	G	-6.5	-5.7	-5.4	-2.4	-6.5	-5.9	-3.8					-2.0	maltose ABC transporter permease
malG	AAK05783.1	G	-4.7	-5.3	-4.3	-2.2	-6.0	-5.6	-2.5						maltose ABC transporter permease
pgmB	SPS12282.1	G	-3.2	-2.8			-2.3	-2.0	-2.5						beta-phosphoglucomutase
rbsD	SPS10141.1	G			-2.3	-2.1				-2.4			-2.6	-2.1	D-ribose pyranase cytoplasmic mutarotase
rbsK	SPS10140.1	G			-2.5	2.0				-2.2			-2.0	-2.1	ribokinase
xylB	QQE99348.1	G					2.5	2.9			2.7				xylulose kinase
xylT	AAK05601.1	G		2.2											D-xylose proton-symporter
xynD	AAK04381.1	G					2.5		2.3	2.1			2.8		endo-1,4-beta-xylanase D
xynT	AAK05604.1	G				2.3									xyloside transporter
yedF	AAK04525.1	G	2.5				2.0	2.0			2.0	2.0			PTS system beta-glucoside-specific transporter; PTS system
yeeA	AAK04526.1	G	-2.8												hypothetical protein ǀ glycoside (trehalose, maltose) hydrolase
yeeB	AAK04528.1	G				-2.1	-2.3		-3.1						sugar hydrolase
yhgE	AAK04856.1	G			2.4	3.8									hypothetical protein
yjbC	AAK04990.1	G									-3.8				hypothetical protein ǀ hexose phosphatase
yngG	AAK05428.1	G									2.2				sugar ABC transporter permease transport and binding protein

ypdA	AAK05595.1	G			3.2				2.3	2.4	2.0	2.4	2.7	sugar ABC transporter substrate binding protein
yqhA	AAK05727.1	G					2.1	-2.4						hypothetical protein ǀ aldose-1-epimerase
yrgI	AAK05812.1	G					2.7	-2.3						hypothetical protein ǀ phosphoglycerate mutase
**Coenzyme transport and metabolism**														
dfpA	GEB08447.1	H								-2.2				phosphopantothenoylcysteine decarboxylase
menB	SPS12340.1	H				3.4								naphthoate synthase
menD	SPS12342.1	H								2.2				2-oxoglutarate decarboxylase menaquinone and ubiquinone
nadD	GFO78988.1	H										2.4		nicotinic acid mononucleotide adenylyltransferase
pyrZ	AAK05445.1	H							2.5			2.2		dihydroorotate dehydrogenase electron transfer subunit
ribA	GFO78927.1	H	2.0											3,4-dihydroxy-2-butanone 4-phosphate synthase
yccH	AAK04324.1	H					2.2				2.5			5'nucleosyl;-tetraphosphatase
ychG	AAK04378.1	H								-2.2				hypothetical protein ǀ Protoporphyrinogen oxidase
yjhB	AAK05043.1	H					3.0							hypothetical protein ǀ 1,4-dihydroxy-2-naphthoate octaprenyltransferase
yuhD	AAK06091.1	H					2.2							hypothetical protein ǀmethyltransferases
**Lipid transport and metabolism**														
accD	SPS12911.1	I	-2.4											acetyl-CoA carboxylase subunit beta
fabZ1	AAK04663.1	I	-2.9											(3R)-hydroxymyristoyl-ACP dehydratase
mvaA	PLW59014.2	I					-3.0			-3.3				hydroxymethylglutaryl-CoA reductase
ydhD	AAK04467.1	I		3.5	3.8					2.8				hypothetical protein
yebA	AAK04504.1	I								-2.8				mevalonate kinase

yrjF	AAK05835.1	I		2.6						hypothetical protein
**Translation, ribosomal structure and biogenesis**										
def	SPS11935.1	J					-4.2			peptide deformylase
infB	SPS12378.1	J		2.4						translation initiation factor IF-2
infC	SPS12498.1	J			-2.3		-4.1			translation initiation factor IF-3
lysS	SPS12107.1	J		-5.4						lysyl-tRNA synthetase class II
mnmA	SPS12635.1	J	-2.0							tRNA-specific 2-thiouridylase MnmA
rnpA	SPS12578.1	J	2.6	2.8	2.2		2.2			ribonuclease P
rplC	SPS12433.1	J				-2.0				50S ribosomal protein L3
rplD	SPS12434.1	J			-2.1		-2.2			50S ribosomal protein L4
rplT	SPS12500.1	J							2.2	50S ribosomal protein L20
rpmE2	SPS12051.1	J		2.9	5.0	2.2	6.5	5.1	3.3	50S ribosomal protein L31 RpmE2
rpmI	SPS12499.1	J		2.2						50S ribosomal protein L35 ribosomal proteins
rpsF	SPS10925.1	J		2.3						30S ribosomal protein S6
rpsG	SPS10759.1	J	-2.6							30S ribosomal protein S7
rpsN2	AAK04986.1	J				-2.2				30S ribosomal protein S14
rpsT	AAK05837.1	J							2.0	30S ribosomal protein S20
serS	SPS12712.1	J	-6.1	-5.8	-5.1	-3.1	-6.7	-6.3	-3.9	seryl-tRNA synthetase
ybgE	GFO78072.1	J		2.4						amidase
yhdC	AAK04831.1	J		2.3						acetyl transferase
ylaG	AAK05199.1	J					-4.3			hypothetical protein
ymdC	AAK05309.1	J		2.1						kanamycin kinase
ynjI	AAK05309.1	J		2.5						hypothetical protein
yqfF	AAK05713.1	J							2.1	spermidine acetyltransferase
yyaL	AAK04104.1	J		2.3						GTP-dependent nucleic acid-binding protein EngD
**Transcription**										

mleR	GFO78800.1	K			-2.2	-2.2					malolactic fermentation system transcription activator
pyrR	SPS10181.1	K			2.2	2.2					bifunctional pyrimidine regulatory protein PyrR
rbsR	AE005176.1	K		-2.4	-2.2						ribose operon repressor LacI-family regulators
rcfA	AAK06101.1	K		-2.0	-2.1						transcriptional regulator CRP/FNR family
rgrA	GEB08621.1	K						-2.5			GntR family transcriptional regulator
rliB	AAK05598.1	K		2.1	2.7			2.1			transcriptional regulator LacI-family regulators
rmaE	AAK05580.1	K			2.4						MarR family transcriptional regulator MarR-family regulator
rmeC	AE005176.1	K				-2.6					transcriptional regulator MerR family of transcription regulators
ybeD	GFO78053.1	K							-2.3		transcriptional regulator TetR/AcrR family
ycfA	NC_004851.1	K				-2.6					transcriptional regulator TetR/AcrR family of transcription regulators
ygfC	AAK04759.1	K		-4.7	-5.3	-4.6	-2.2	-6.0	-5.6	-3.1	transcriptional regulator TetR/AcrR family
yjaH	AAK04983.1	K			3.1						hypothetical protein
yjaJ	AAK04985.1	K			2.7						transcriptional regulator belongs to LytR family
yliB	AE005176.1	K				-2.3					hypothetical protein
ynaB	AAK05369.1	K	2.4	2.0	2.9						transcriptional regulator
yohC	AAK05547.1	K							-2.3		transcriptional regulator belongs to TetR/AcrR family
yqbH	AAK05674.1	K				2.4	2.5			3.0	transcriptional regulator
ytgE	AAK05996.1	K						2.3			hypothetical protein
yucF	AAK06047.1	K		2.2	3.4						hypothetical protein

yveF	AAK06157.1	K		2.2	2.3	2.8	-2.1		2.5				hypothetical protein
**Replication, recombination and repair**													
dnaB	SPS12362.1	L				2.5							hypothetical protein
dnaI	WP_060416476.1	L			-3.4	-4.9			-3.4	-3.7	-2.2		primosomal protein DnaI
dnaN	PS10614.1	L						-3.2					DNA polymerase III subunit beta
dnaQ	AAK05080.1	L						-2.1					DNA polymerase III subunit epsilon
mutM	SPS12735.1	L			2.6	2.8	-2.2						formamidopyrimidine-DNA glycosylase
ogt	PLW60489.1	L						2.0	-3.5				6-O-methylguanine DNA methyltransferase
polC	AAK06222.1	L				2.4							DNA polymerase III PolC
recJ	SPS10369.1	L							-2.1	-2.1			ssDNA-specific exonuclease RecJ
recO	SPS12671.1	L				2.6							DNA repair protein RecO
sbcC	AAK05419.1	L									2.0		ATP-dependent dsDNA exonuclease degradation of DNA
scpA	AE005176.1	L					-2.4						segregation and condensation protein A
tag	AAK05220.1	L			-2.5				2.4				DNA-3-methyladenine glycosidase
tra1077C	AAK04470.1	L			6.4	6.8	8.1	3.0	7.0	6.7	5.4		IS1077C transposase
tra1077D	AAK04737.1	L							-2.1				IS1077D transposase
tra1077E	AAK04922.1	L							-3.0				IS1077E transposase
tra904A	AAK04151.1	L	3.8	5.3								3.4	transposase of IS904A
tra904B	AAK04235.1	L	3.7	4.8								2.2	transposase of IS904B
tra904D	AAK04468.1	L	3.8	4.9		3.0			3.1			2.4	transposase of IS904D
tra904E	AAK04735.1	L	3.5	4.2								2.2	transposase of IS904E
tra983A	AAK04779.1	L							-3.6				transposase of IS983A
trpB	SPS11791.1	L									3.0		tryptophan synthase subunit beta
xseA	GEB09426.1	L							5.0				exodeoxyribonuclease VII large subunit
yafG	AAK04149.1	L		2.0	2.2	3.2							hypothetical protein
yafI	AAK04152.1	L	3.5	3.9									hypothetical protein

ybdK	AAK04234.1	L	2.0	3.5	2.7					2.7	2.0			hypothetical protein transposon related function
ygcE	AAK04738.1	L				2.9	4.4							hypothetical protein
yidF	AAK04921.1	L		3.6	3.9								2.1	hypothetical protein
ysiD	AAK05915.1	L					2.7	-2.2						hypothetical protein
ytfB	AAK05980.1	L					2.9							hypothetical protein
**Cell wall/membrane/envelope biogenesis**														
dacB	AAK05048.1	M					2.9							D-alanyl-D-alanine carboxypeptidase murein sacculus and peptidoglycan
dapA	SPS10159.1	M				2.3		2.4	2.3			2.9		dihydrodipicolinate synthase
ftsQ	AAK05686.1	M										2.1		hypothetical protein
glmS	WP_058223713.1	M	2.0											glucosamine--fructose-6-phosphate aminotransferase
murB	SPS10276.1	M					2.5							UDP-N-acetylenolpyruvoylglucosamine reductase
pbp2A	AIS04644.1	M					2.9							penicillin-binding protein 2a
plpB	AAK04417.1	M							2.2					outer membrane lipoprotein
rgpE	AAK04304.1	M					2.8	-2.2						glycosyltransferase
ycbF	AAK04311.1	M					2.5							LPS biosynthesis protein
ycbG	AAK04312.1	M					2.1							LPS biosynthesis protein
yibC	AAK04903.1	M								-3.9				hypothetical protein
**Cell motility**														
acmA	AAK04370.1	N					2.22	-2						autolysin cell wall hydrolase
**Posttranslational modification, protein turnover, chaperones**														
ahpC	SPS12269.1	O					-2.5	-2.2	3.2					alkyl hydroperoxide reductase
gcp	SBW29465.1	O								-4.7				DNA-binding/iron metalloprotein/AP endonuclease
osmC	PLW60135.1	O				-6.5	-5.7	-5.4	-2.4	-6.5	-5.9	-4.9		osmotically inducible protein

pflA	QOK49714.1	O					2.8	-2.0			pyruvate-formate lyase activating enzyme
trxB2	AAK05745.1	O			-2.2	2.2			-3.4		thioredoxin reductase
ydiE	AAK04480.1	O				2.5	3.6	-2.4	2.6		hypothetical protein
**Inorganic ion transport and metabolism**											
amtB	AAK05690.1	P		4.1							ammonium transporter
cbiO	SBW29503.1	P	2.2			5.8		2.2		3.8	cobalt importer protein; ATP-binding subunit
dpsA	GFO79963.1	P		-2.0							non-heme iron-binding ferritin
fhuB	PLW60363.1	P							-2.1		ferrichrome ABC importer permease
mtsA	AAK05414.1	P	-2.7	-3.4							manganese ABC importer
mtsB	AAK05416.1	P	-2.2	-3.2							manganese ABC importer
mtsC	AAK05415.1	P	-2.9	-4.3			-2.3				manganese ABC importer permease
pacL	AAK04776.1	P							2.3		magnesium-transporting ATPase
yafB	AAK04148.1	P				2.1	3.0				sulfate permease, MFS superfamily
ydaE	AAK04406.1	P							2.3		Co/Zn/Cd efflux system component
yigC	AAK04943.1	P					2.5				arsenate reductase
yoaB	SPS12037.1	P					2.7				magnesium-transporting ATPase
yogJ	AAK05544.1	P					2.2				Fe/Co/Zn/Cd efflux system component
yuhE	AAK06092.1	P					2.6				copper homeostasis protein
yuiA	AAK06092.1	P					2.6				heavy metal-transporting ATPase
zitQ	AAK06212.1	P					3.1	-2.3			zinc ABC importer ATP binding protein
**Secondary metabolites biosynthesis, transport and catabolism**											
dltA	WP_058205708.1	Q				-2.9		-3.0		-2.0	D-alanine--poly(phosphoribitol) ligase subunit 1
dltC	QQE99144.1	Q				-2.1		-3.6			D-alanine--poly(phosphoribitol) ligase subunit 2
fabF	SPS12386.1	Q		-2.3							3-oxoacyl-ACP synthase fatty acid and phospholipid metabolism

fabG	QNL91178.1	Q		-2.3										3-ketoacyl-ACP reductase
ykaF	AAK05079.1	Q									-2.4	-2.3		hypothetical protein
**General function prediction**														
comEC	AAK05870.1	R											2.7	hypothetical protein
engA	KWT47845.1	R						2.8						GTP-binding protein EngA
yagB	AIS02642.1	R						2.1						hypothetical protein
ybhD	AAK04270.1	R					-2.2		-2.3					hypothetical protein
yceE	AAK04342.1	R											4.0	hypothetical protein
ycfF	SBW29425.1	R									-3.5	-2.3		hypothetical protein
ydhB	AAK04465.1	R									-2.7			hypothetical protein
yeaA	AAK04493.1	R										-3.0		hypothetical protein
yfgH	AAK04668.1	R						2.8						hypothetical protein
yheB	AAK04833.1	R											2.4	hypothetical protein
yhhD	AAK04860.1	R				2.5								hypothetical protein
yihF	AAK04954.1	R									-2.3			hypothetical protein
ykiF	AAK05173.1	R								2.6				hypothetical protein
ymdE	AAK05315.1	R								-3.2				hypothetical protein
yqeL	AAK04319.1	R					-2.2					4.6		GTP-binding protein YqeH
yqfE	AAK05712.1	R						2.5						hypothetical protein
yqgA	AAK05716.1	R						2.4						hypothetical protein
yrjE	AAK05834.1	R						2.6						transport permease
ysbA	AAK05851.1	R	2.1	2.3	2.9	2.7								hypothetical protein
yseA	AAK05879.1	R									-2.6			hypothetical protein
ytaB	AAK05932.1	R					2.2	2.9						hypothetical protein
ythC	AAK06004.1	R				2.9								hypothetical protein
ywfD	AAK06275.1	R								2.6				hypothetical protein
yxfC	AAK06364.1	R						2.2						hypothetical protein
**Function unknown**														

L200001		S			-4.1		-2.2				-2.6		hypothetical protein similar to prophage bIL312 protein 12
L200003		S			2.5	3.5							hypothetical protein
L200005		S			2.5	2.2							hypothetical protein
L200032		S							2.3				hypothetical protein
L200034		S				2.8							hypothetical protein
L200039		S							-2.7				hypothetical protein similar to bacteriophage bIL285
L200065		S		2.4		2.7	-2.2		2.1				hypothetical protein
L200074		S	2.9	2.4	2.0	2.5	-2.3	-2.4	2.1				hypothetical protein
L200078		S			2.1		2.7	4.5			4.4	3.7	sodium:solute symporter, former pseudogene
yacI	AAK04112.1	S			2.3								hypothetical protein
yaiG	AAK04182.1	S			2.7	3.2	-2.2		2.0				hypothetical protein
yaiJ	BAL52160.1	S		3.3					-3.2				hypothetical protein
ybaC	AAK04199.1	S			-3.8		2.6			6.1	-2.4		hypothetical protein
ybbB	SBW29265.1	S				-2.2	-2.6		-4.3	-2.3			hypothetical protein
ybcG	AAK04218.1	S				2.6							hypothetical protein
ybfA	AAK04251.1	S									3.1		hypothetical protein
ybgB	GFO78053.1	S				2.6							hypothetical protein
ybjD	AAK04293.1	S			2.6	2.5							hypothetical protein
ycbA	AAK04306.1	S							-2.2				hypothetical protein
ycdB	AAU43207.1	S						-2.1					hypothetical protein
ycjG	AAK04395.1	S				2.6							hypothetical protein
ydbD	AAK04412.1	S		2.0						2.2			hypothetical protein
yedA	AAK04521.1	S				2.8							hypothetical protein
yfdG	AAK04648.1	S			2.1								hypothetical protein
yfhH	AAK04680.1	S			-3.6						-2.3		hypothetical protein

yfiH	AAK04692.1	S					2.2						hypothetical protein
yfiI	AAK04694.1	S					3.0	-2.1					hypothetical protein
ygaD	AAK04713.1	S				-2.4							hypothetical protein
ygfE	AAK04761.1	S									-2.4		magnesium importer and exporter
yghD	AAK04774.1	S										2.3	hypothetical protein
yhbH	AAK04810.1	S								-4.0			hypothetical protein
yhhE	AAK04861.1	S					2.8						hypothetical protein
yhjA	AAK04881.1	S	-2.2							-2.7			hypothetical protein
yibD	AAK04904.1	S										2.2	hypothetical protein
yiiF	AAK04961.1	S										3.6	transporter
yjaE	AAK04980.1	S	3.2										hypothetical protein transmembrane protein
yjhA	AK05042.1	S									-2.7		hypothetical protein
yjjA	AAK05061.1	S									-3.3		hypothetical protein
ykbC	AAK05084.1	S				2.7	4.3						hypothetical protein
ylfI	AAK05242.1	S										2.5	hypothetical protein
ylgB	AAK05246.1	S								-2.2		2.1	hypothetical protein
yliD	AAK05268.1	S		2.1	3.7			-2.1					hypothetical protein
ymeA	AAK05317.1	S			3.5							3.1	hypothetical protein
ymhC	AAK05348.1	S				3.1	-2.5						hypothetical protein
ynbD	AAK05394.1	S								-2.4			hypothetical protein
ynjH	AAK05458.1	S				4.4	6.7	6.4	2.3	6.2	6.2	3.4	hypothetical protein
yogI	AAK05543.1	S	-2.3										phosphate-starvation-inducible protein PsiE
ypgH	AAK05624.1	S				-5.3						-3.3	hypothetical protein
yphH	AAK05632.1	S							2.7				hypothetical protein
yphK	AAK05635.1	S				-2.3				-2.8			hypothetical protein
ypiH	AAK05645.1	S									2.1		hypothetical protein

yqeA	AAK05695.1	S					-3.7		2.0				-2.3	hypothetical protein
yraD	AAK05753.1	S					2.8	4.4						hypothetical protein
yraF	AAK05756.1	S									-2.1			hypothetical protein
yrbB	AAK05760.1	S							-2.3	-2.2	-2.1			hypothetical protein
yrbH	AAK05766.1	S						-2.6		3.2				hypothetical protein
yreD	AAK05787.1	S	3.3	3.8	4.9	3.8								hypothetical protein
yrgE	AAK05807.1	S									2.4			hypothetical protein
yriB	AAK05827.1	S					2.1		2.4				2.9	hypothetical protein
ysbB	AAK05852.1	S						2.8						hypothetical protein
ysbC	AAK05853.1	S			2.0	2.1								hypothetical protein
yseD	AAK05883.1	S					2.7	3.0	-2.2		2.4			hypothetical protein
yshB	AAK05909.1	S						2.7	-2.1					hypothetical protein
ysjF	AAK05925.1	S						3.0	-2.2					hypothetical protein
ytcA	AAK05950.1	S							-2.6	-2.2				hypothetical protein
yuaB	AAK06021.1	S					2.3						3.0	hypothetical protein
yuiE	AAK06109.1	S											2.6	hypothetical protein
yujG	AAK06119.1	S											4.4	hypothetical protein
yvcA	AAK06139.1	S					2.2	2.3						hypothetical protein
yveG	AAK06158.1	S					2.1							hypothetical protein
yviA	AAK06207.1	S					-3.8				-2.2		-2.5	hypothetical protein
yxcE	AAK06341.1	S						2.3						hypothetical protein
**Signal transduction mechanisms**														
ytaD	AAK05934.1	T				2.3					2.8	2.1		tyrosine phosphatase translation
ythA	AAK06002.1	T				2.3								hypothetical protein
ythB	AAK06003.1	T				2.7	-2.1	-3.0		4.1	-2.2			hypothetical protein
**Intracellular trafficking, secretion, and vesicular transport**														
clpP	AIS03218.1	U										-2.8		ATP-dependent Clp protease proteolytic subunit

comGA	AAK06221.1	U		2.7	3.0	-2.1					protein ComGA competence protein
sipL	GFO80241.1	U			2.5						signal peptidase I
**Defence mechanisms**											
blt	AAK04221.1	V						2.2			multidrug efflux transporter multidrug resistance
hsdS	AAK57812.1	V		-3.0		-2.6				-2.1	type I restriction enzyme specificity protein
lmrA	QQF01231.1	V			2.6						multidrug resistance ABC transporter ATP-binding protein
lmrP	QQF00039.1	V						2.4			multidrug efflux MFS transporter
napC	GFO78231.1	V			3.6						multidrug-efflux transporter multidrug resistance
ycfC	AAK04353.1	V		3.0							ABC transporter permease
ydaG	AAK04408.1	V						2.3			multidrug resistance ABC transporter ATP-binding protein
ypbC	AAK05582.1	V	3.3							3.1	MATE family efflux transporter
ypgD	AAK05620.1	V		-3.4		-2.8				-2.2	multidrug resistance ABC transporter ATP-binding protein
yvhA	AAK06200.1	V			2.2						MATE family efflux transporter
ywiG	AAK06301.1	V	2.1							2.0	ABC-type multidrug transport system, ATPase component
**Mobilome:prophages, transposons**											
bIL30901		X		-2.3							integrase prophage bIL309 protein 01
bIL30909		X			2.8						prophage bIL309 protein 09
bIL30911		X			3.2						recombinase prophage bIL309 protein 11
bIL30913	GCF_000842505.1	X							-2.1		replisome organizer prophage bIL309 protein 13
bIL30914		X			2.3						DNA replication protein prophage bIL309 protein 14
bIL30922		X		2.5		2.2	2.3			2.8	prophage bIL309 protein 22

bIL30923		X			-2.1	-2.1				prophage bIL309 protein 23
bIL30928		X	-5.4	-2.5		2.2		6.7	-3.4	prophage bIL309 protein 28
bIL30929		X	3.0							prophage bIL309 protein 29
bIL30932		X	2.3	3.3			2.4			prophage bIL309 protein 32
bIL30936		X	-2.3	-3.9	-2.2		-3.7	-3.5		prohead protease prophage bIL309 protein 36
bIL30938		X					2.2			prophage bIL309 protein 38
bIL30942		X		-2.2	-2.1					small major structural protein prophage bIL309 protein 42
bIL30943		X		2.3						prophage bIL309 protein 43
bIL30945		X				-2.1				choline ABC transporter permease and substrate binding protein
bIL30949		X				2.1				muramidase prophage bIL309 protein 49
choS	AAK04946.1	X				-2.1				prophage bIL309 protein 45 tail component
pi206		X		3.0						prophage bIL285 protein 06
pi215		X		2.0						prophage bIL285 protein 15
pi240		X		-3.0	-2.1		-2.0			prophage bIL285 protein 40
pi301		X							2.2	prophage bIL286 protein 01
pi308		X	2.6	4.0						prophage bIL286 protein 08
pi319	GCF_000845385.1	X		2.2						prophage bIL286 protein 19 tail component
pi325		X		2.5		-3.2				head-tail joining protein prophage bIL286 protein 25
pi333		X	2.7	2.8	-2.4		2.4			prophage bIL286 protein 33
pi336		X					2.0			prophage bIL286 protein 36
pi338		X		2.0	-2.2					prophage bIL286 protein 38
pi339		X	2.8		2.5	2.4			2.9	prophage bIL286 protein 39
pi341		X					2.4			prophage bIL286 protein 41

pi346		X						2.9		-2.3				DNA replication protein prophage bIL286 protein 46
pi348		X											2.3	single strand binding helix destabilising protein prophage bIL286 protein 48
pi349		X									3.8			prophage bIL286 protein 49
pi360		X									2.8			integrase prophage bIL286 protein 60
ps101		X				3.1				2.3				prophage bIL310 protein 01
ps105		X					2.8	3.7						DNA primase prophage bIL310 protein 05
ps106		X					2.1	3.0						prophage bIL310 protein 06
ps107		X				2.4	2.2	2.8			2.0			prophage bIL310 protein 07
ps109		X					2.6	4.2						prophage bIL310 protein 09
ps115		X									-2.2			prophage bIL310 protein 15 transcription regulator
ps116		X					-3.5		-3.0				-2.3	prophage bIL310 protein 16
ps210	GCF_000838045.1	X									2.6			prophage bIL312 protein 10
ps211		X						2.3						prophage bIL312 protein 11
ps216		X				2.1								prophage bIL312 protein 16
ps303		X	3.2	2.4										prophage bIL311 protein 03
ps304		X	2.4	2.4										prophage bIL311 protein 04
ps305		X	2.2											prophage bIL311 protein 05
ps306		X			2.5	3.7					2.0			prophage bIL311 protein 06
ps311		X									-4.0			prophage bIL311 protein 11
ps315		X							-2.5	-2.5				prophage bIL311 protein 15
